# Examining barriers to healthcare access and utilization of antenatal care services: evidence from demographic health surveys in sub-Saharan Africa

**DOI:** 10.1186/s12913-021-06129-5

**Published:** 2021-02-06

**Authors:** Bright Opoku Ahinkorah, Edward Kwabena Ameyaw, Abdul-Aziz Seidu, Emmanuel Kolawole Odusina, Mpho Keetile, Sanni Yaya

**Affiliations:** 1 School of Public Health, Faculty of Health, University of Technology Sydney, Sydney, NSW Australia; 2 Department of Population and Health, College of Humanities and Legal Studies, University of Cape Coast, Cape Coast, Ghana; 3 Department of Demography and Social Statistics, Federal University, Oye, Ekiti Nigeria; 4 Population Studies and Demography, University of Botswana, Gaborone, Botswana; 5 School of International Development and Global Studies, Faculty of Social Sciences, University of Ottawa, 120 University Private, Ottawa, ON K1N 6N5 Canada; 6 The George Institute for Global Health, Imperial College London, London, UK

**Keywords:** Health services, Utilization, Access, Antenatal care, Health care, Disparities, Inequalities, Sub-Saharan Africa; Global Health; public health

## Abstract

**Background:**

Antenatal care utilization is one of the means for reducing the high maternal mortality rates in sub-Saharan Africa. This study examined the association between barriers to healthcare access and implementation of the 2016 WHO antenatal care services model among pregnant women seeking antenatal care in selected countries in sub-Saharan Africa.

**Methods:**

This study considered only Demographic and Health Survey data collected in 2018 in sub-Saharan Africa. Hence, the Demographic and Health Survey data of four countries in sub-Saharan Africa (Nigeria, Mali, Guinea and Zambia) were used. A sample of 6761 from Nigeria, 1973 from Mali, 1690 from Guinea and 1570 from Zambia was considered. Antenatal care visits, categorized as < 8 visits or ≥8 visits, and time of the first antenatal care visit, categorized as ≤3 months or > 3 months (as per the WHO recommendations) were the outcome variables for this study. Both descriptive statistics and ordinal logistic regression were used to analyze the data. Crude odds ratios (cOR) and adjusted odds ratios (aOR) and *p*-values < 0.05 were used for the interpretation of results.

**Results:**

With timing of antenatal care visits, getting money needed for treatment (aOR = 1.38, 95% CI = 1.03–1.92) influenced early timing of antenatal care visits in Mali whereas getting permission to visit the health facility (aOR = 1.62, 95% CI = 1.15–2.33) motivated women to have early timing of antenatal care visits in Guinea. We found that women who considered getting money needed for treatment as not a big problem in Nigeria were more likely to have the recommended number of antenatal care visits (aOR = 1.38, 95% CI= 1.11–1.73). On the contrary, in Guinea, Zambia and Mali, getting permission to visit health facilities, getting money for treatment, distance to the health facility and not wanting to go alone were not barriers to having ≥ 8 antenatal care visits.

**Conclusion:**

Our study has emphasized the role played by barriers to healthcare access in antenatal care utilization across sub-Saharan African countries. There is the need for governmental and non-governmental organizations to ensure that policies geared towards improving the quality of antenatal care and promoting good interaction between health care seekers and health care providers are integrated within the health system.

## Background

Maternal mortality is one of the issues of public health concern globally [1, 2]. As a result, the international community has always laid emphasis on ensuring a reduction in maternal mortality. For example, Goal 5 of the Millennium Development Goals (MDGs) was aimed at reducing maternal mortality rate by 75% by the year 2015 [3]. While a significant decline occured in maternal mortality rates in many countries globally, this target was not achieved, as maternal mortality rate reduced only by 44% globally by the end of 2015 [4, 5]. The MDGs were followed by the Sustainable Development Goals (SDGs), which also aimed at improving women’s health through the reduction of maternal mortality. Specifically, target 3.1 of the SDGs aims at reducing the global maternal mortality ratio to less than 70 per 100,000 live births by the year 2030 [6, 7]. Despite this, there is still high maternal mortality rate globally, with countries in sub-Saharan Africa (SSA) disproportionately affected [6]. With a current maternal mortality rate of 351 per 100, 000 live births, SSA has the highest maternal mortality rate globally [8]. Maternal mortality rate in Nigeria, Mali, Guinea and Zambia are 917, 562, 576 and 213 per 100, 000 live births respectively [9].

Antenatal care (ANC) utilization has been regarded as one of the means for reducing the high maternal mortality rates in SSA [10, 11]. Adequate and timely ANC provides the opportunity for essential health task such as health promotion, screening and diagnosis, and disease prevention [12, 13]. It is a key health care tool that helps to reduce the risk of stillbirths, preterm labor and pregnancy-related complications [14]. Therefore, it is essential for women to receive adequate and timely ANC services for positive experience during pregnancy.

Nonetheless, in SSA, 69% of pregnant women have at least one ANC visit, more than in South Asia, at 54%. Coverage for ANC is usually expressed as the proportion of women who have had at least one ANC visit. However, coverage of at least four ANC visits is lower at 44%, as shown on the country profiles. Trends indicate slower progress in SSA than in other regions, with an increase in coverage of only 4 % during the past decade [15].

To reduce the high rates of pregnancy-related mortality and to ensure that the SDG target of reducing the global maternal mortality ratio to less than 70 per 100,000 live births by the year 2030 is met, the WHO in November 2016 came up with a guideline and recommendations on ANC and this included an increase in recommended antenatal visits from 4 to 8 contacts or more. To enhance quality, appropriate timing and maximum impact of care, the WHO also introduced the ANC model which specifies that pregnant women should have their first ANC contact in the first 12 weeks of gestation, with subsequent contacts at 20, 26, 30, 34, 38 and 40 weeks of gestation. The model also emphasized the need to ensure good attitude, comprehensive and person-centered care at each contact and the provision of timely and relevant information to pregnant women [13].

To provide evidence on how these new guidelines, recommendations and models of focused ANC (FANC) are working and to assess some of the challenges associated with these, it is essential that a study is conducted on current ANC utilization in a region like SSA which has constantly recorded highest prevalence of maternal mortality globally. Based on evidence that several barriers to healthcare access influence maternal healthcare services utilization in SSA [16–18] and the new WHO recommendations on ANC, this study examined the association between barriers to healthcare access and implementation of the 2016 WHO ANC model among pregnant women seeking ANC in selected countries in SSA.

## Methods

### Study setting

SSA is the portion of the continent of Africa that lies south of the Sahara. According to the United Nations, the region consists of all African countries and territories that are fully or partially south of the Sahara and it geographically consists of 46 countries, including Nigeria, Guinea, Mali and Zambia [19]. SSA is home to over 500 million women who account for about half of the continent’s population and 14% of the female population worldwide and approximately 47% of them are of reproductive age (15–49, [20]). Despite an overall improvement in maternal survival and a 45% decrease in maternal mortality rate globaly since 1990, women in SSA continue to bear an unacceptable health burden [21]. This has often been attributed to a number of factors including the lack of universal access to essential services and interventions and maternal health related services are not an exception [22]. Hence, millions of women in SSA are not accessing maternal health services, and undergo their pregnancies and childbirths outside the health system and this explains sub-Saharan African countries' excessive share of the global burden of disease and death, particularly as it relates to maternal and reproductive health [23].

### Data source

This study only considered Demographic and Health Survey (DHS) data collected in 2018 in SSA. The inclusion criteria were such that all countries with datasets that were more than a year after the November 2016 WHO new guidelines for ANC services were considered for analyses. We used all datasets available for SSA countries collected in the year 2018 or later considering 9–10 months gestation period of pregnancy. Thus, the DHS data of Nigeria, Mali, Guinea and Zambia were used. The DHS is a standardized cross-sectional survey carried out in over 90 low-to-middle income countries with the aim of providing current estimates on a number of health indicators and to track countries' progress on the SDGs.

### Data collection

The surveys employ a two-stage stratified sampling in sampling the research participants, where countries are grouped into urban and rural areas. The first stage involves the selection of clusters usually called enumeration areas (EAs) and the second stage consists of the selection of household for the survey. To ensure consistency in data collection across countries, the DHS uses a standard questionnaire comparable across countries for data collection, and the questionnaire is often translated into the major local languages of the countries involved [24]. To ensure validity of the translated questionnaires, the DHS reports that the translated questionnaires together with the version in English are pretested in English and the local dialect. After that, the pretest field staff actively discussed the questionnaires and made suggestions to modify all versions. Following field practice, a debriefing session is held with the pretest field staff, and modifications to the questionnaires were made based on lessons drawn from the exercise. Details of the sampling methods, procedures and implementation can be found on the DHS website in each country final report [25–28]. Table 1 provides detailed information on the period of data collection and sample sizes for each eligible country. To assess factors associated with the WHO recommendations (ANC visit ≥8 and ANC timing ≤3 months), we extracted information on all currently married women, who gave birth in the last 0–12 months prior to the month of interview, responded to questions on ANCvisits and timing and had complete response for all variables considered.

**Table 1:** Country and sample size details

Country	Survey (data collection) period	Total women (aged 15–49 years) interviewed	Sample size by design^a^	Selected women sample^b^	% of completed responses
Nigeria	August 14–December 29, 2018	41,821	6857	6761	98.5
Mali	August 6–November 18, 2018	10,519	2049	1973	96.3
Guinea	March 27–June 28, 2018	10,874	1708	1690	98.9
Zambia	July 17–January 24, 2019	13,683	1637	1570	95.9

^a^Sample size by design are women aged 15–49 currently married, who gave birth in the last 0–12 months prior to the month of interview and responded to questions on antenatal care visits and timing ^b^Selected women sample are women with complete response for all variables considered

#### Variables

##### Outcome variables

For this study, two ANC variables (timing of ANC visits and number of ANC visits) were investigated:

i) Timing of ANC visits was defined as the period during pregnancy when the first ANC was sought. It was derived from the question ﻿ “How many months pregnant were you when you first received ANC for this pregnancy? The responses from this question were categorized as ≤3 months or > 3 months. ANC visits that occurred ≤3 months are also known as early ANC visits [13].
ii) Number of ANC visits in this study was defined as the number of times a pregnant woman received care during pregnancy. To derive this variable, the DHS asked a question “How many times did you receive ANC during this pregnancy?. The responses from this question were categorized as < 8 visits or ≥8 visits. ≥8 visits is the recommended number of ANC visits by WHO [13].

##### Independent variables

Four key variables, that measure barriers to healthcare access were considered as key explanatory variables. These were getting permission to visit health facilities, getting money for treatment, distance to the health facility and not wanting to go alone. Each of these variables were categorized into ‘big problem’ and ‘not a big problem’. Our interest was to find out whether those who considered each of the barriers as ‘not a big problem’ will attend the ≥8 visits and receive their first ANC ≤3 months during pregnancy. Apart from the key independent variables, other factors considered relevant with ANC utilization were also included in the analyses as covariates. These include age of women at childbirth (≤19, 20–24, 25–29, 30–34, 35–39, ≥40), residence (urban vs rural), religion (Christians, Muslims and others), birth order (1–2, 3–4, ≥5), pregnancy intention (no vs yes) and polygyny (monogamous, polygamous as first wife, polygamous as second wife or latter). Other relevant variables selected were health insurance coverage (no vs yes), wealth quintile (poorest, poorer, middle, richer, richest), husband’s highest educational level (no education, primary, secondary, tertiary) and the difference in age between husband and wife (wife older or same age with husband, husband 1–5 years older, husband 6–10 years older, husband more than 10 years older).

The selection of these variables was influenced by the Health Care Services Utilisation Model by Andersen and Newman [29]. The model is a behavioural model that explains the conditions that either promote or hinder the utilisation of health care services [29]. This model identified three main conditions or factors that influence an individual to or not use a health care service. These factors are the Predisposing factors, Enabling factors and Need for care factors.

Predisposing factors refer to the demographic, social structure and health belief characteristics. The demographic characteristics of the individual that affect the decision to use or not use a health care service. Social Structure consist of factors surrounding education and occupation and health belief factors consist of values, attitudes of health care service providers, and knowledge about health [29]. In this study, the predisposing factors were age of women at childbirth, religion, birth order, polygyny, husband’s highest educational level and the difference in age between husband and wife.

Enabling factors are the resources or means that is available to an individual to seek health care services. Enabling factors are measured at the household level, thus, the availability of income and the community level, thus, the availability and location of health care facilities in the community [29]. In this study, the enabling factors included getting permission to visit health facilities, getting money for treatment, distance to the health facility, not wanting to go alone, residence and wealth quintile.

**Fig. 1:**
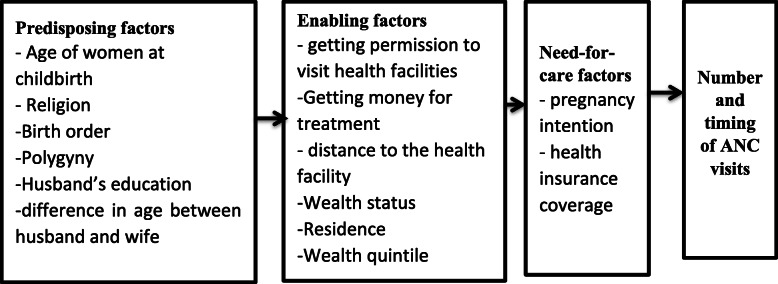
Conceptual Framework

Need for care factors refer to how an individual perceives his own general health and functional condition, as well as their familiarity with the signs and symptoms of ill health, agony and concerns about their health [29]. The need for care factors is influenced by the predisposing factors and the enabling factors of an individual. In this study, the need for care factors included pregnancy intention and health insurance coverage (Fig. 1).

#### Data analyses

Statistical analyses were performed using Stata 14.0. Data on respondents who had complete information and responses for all variables considered in this study were used for the analyses. Data were first summarized by presenting the frequency distributions of the variables using descriptive statistics of frequency and percentages for each of the four countries considered in this study. Next, number of women with at least 8 ANC visits and early timing of first ANC visit (≤3 months of gestation) by the four measures of barriers to healthcare access were presented as frequency distributions and corresponding 95% confidence intervals (CI) at *p*< 0.05. This was followed by a crude ordinal logistic regression to ascertain the relationship between number and timing of ANC and the independent variables, with the results presented as crude odds ratios (cOR). The final step of the analyses involved the use of an ordinal logistic regression to examine the relationship between number and timing of ANC and the variables for measuring barriers to healthcare access, while adjusting for other covariates, with the results presented as adjusted odds ratios (aOR). We used weighting, clustering and stratification to adjust for the complex survey design. *P*-values < 0.05 were used for interpretation of results.

#### Ethical consideration

Ethical permissions were not required for this study since we used DHS datasets already publicly available. The DHS reports that ethical procedures were the responsibility of the institutions that commissioned, funded, or managed the surveys. All DHS surveys are approved by Inner City Fund (ICF) international as well as an Institutional Review Board (IRB) in respective countries to ensure that the protocols are in compliance with the U.S. Department of Health and Human Services regulations for the protection of human subjects. In compliance with the declaration of Helsinki on human research, the consent for participation were duly obtained from respondents before data collection. The dataset for countries where DHS is conducted can be accessed for free after due permission from the DHS Measures.

## Results

### Description of study sample

This study considered a weighted percentage of currently married women who gave birth within 0–12 months prior to the survey and had complete cases on all the variables of interest as eligible respondents. The sample sizes ranged from 1526 women in Zambia to 6761 women in Nigeria (see Table 1). Most of the women had < 8 ANC visits that ranged from 82.2% in Nigeria to 98.5% in Zambia as well as first ANC visit > 3 months of gestation, with variations ranging from 61.5% in Zambia to 82.4% in Nigeria.

The distribution of women characteristics such as age at birth, place of residence, religion, birth order, pregnancy intention, polygyny, wealth quintiles, health insurance, husbands’ level of education and difference in age between husband and wife varied across countries. In Nigeria (29.1%), Mali (26.2%) and Guinea (27.3%), most of the women gave birth to their children at age 25–29 whereas in Zambia, the age at childbirth for most of the women was at age 20–24 (37.6%). In all the four countries, most of the women lived in rural areas. The dominant religion for a higher number of the women in Nigeria (65.4%), Mali (92.6%) and Guinea (88.9%) was Islam, while most of the women in Zambia were Christians (97.5%). In Nigeria and Zambia, most of the women had ≥5 birth order (36 and 38.1% respectively) whereas in Guinea and Zambia, the majority of the women had 1–2 children (33.9 and 38.1% respectively).

In all four countries, majority of the women whose pregnancies were planned had monogamous marriages. Whereas most women in Nigeria and Mali were in the poorer wealth quintiles (23.1 and 21.9% respectively), majority of the women in Guinea and Zambia were in the poorest wealth quintile (26.7 and 25.8% respectively). Majority of women in all the four countries were not covered by health insurance. Whereas in Nigeria (37.2%), Mali (70.8%) and Guinea (74.2%), most of the women’s husbands had no education, majority of the husbands of women in Zambia had secondary education (47.5%). In terms of difference in age between husband and wife, most of the women in Nigeria (38.2%), Mali (43.6%) and Guinea (51.0%) had husbands who were > 10 years older, while in Zambia, majority of women had husbands who were 1–5 years older (54.1%) (see Table 2).

**Table 2:** Descriptive Characteristics of currently married women with most recent birth in Nigeria, Guinea, Mali, Zambia (DHS 2018)

Variable	Nigeria (***N***=6761)	Mali (***N***=1973)	Guinea (***N***=1690)	Zambia (***N***=1570)
	n(%)	n (%)	n (%)	n (%)
***Demographic***				
**Number of antenatal visits**				
< 8	5555(82.2)	1903(96.5)	1642(97.2)	1547(98.5)
≥ 8	1206(17.8)	69(3.5)	48(2.8)	23(1.5)
**Time of first antenatal visit**				
≤ 3 months of gestation	1188(17.6)	701(35.5)	445(26.3)	604(38.5)
> 3 months of gestation	5573(82.4)	1272(64.5)	1245(73.6)	966(61.5)
**Age at child birth (years)**				
< 19	614(9.1)	246(12.5)	233(13.8)	171(10.9)
20–24	1530(22.6)	498(25.2)	357(21.1)	420(37.6)
25–29	1966(29.1)	516(26.2)	461(27.3)	371(23.6)
30–34	1402(20.7)	362(18.4)	314(18.6)	307(19.5)
35–39	848(12.5)	254(12.9)	211(12.5)	211(13.4)
≥40	399(5.9)	97(4.9)	114(6.8)	90(5.8)
**Place of residence**				
Urban	2511(37.1)	391(19.8)	432(25.6)	513(32.7)
Rural	4250(62.9)	1582(80.2)	1258(74.4)	1057(67.4)
**Religion**				
Christians	2298(34.0)	62(3.1)	158(9.3)	1531(97.5)
Muslims	4424(65.4)	1827(92.6)	1502(88.9)	13(0.8)
Others	40(0.6)	84(4.3)	30(1.8)	26(1.7)
**Birth order**				
1–2	2384(35.3)	643(32.6)	573(33.9)	598(38.1)
3–4	1946(28.8)	578(29.3)	565(33.4)	460(29.3)
≥5	2430(36.0)	753(38.1)	552(33.7)	511(32.6)
**Pregnancy wanted**				
No (later/no more)	764(11.3)	347(17.6)	272(16.1)	557(35.5)
Yes	5997(88.7)	1626(82.4)	1418(83.9)	1013(64.5)
**Polygyny**				
Monogamous	4728(69.9)	1303(66.1)	1038(61.4)	1397(89.0)
Polygamous as first wife	743(11.0)	254(12.9)	220(13.0)	69(4.4)
Polygamous as 2nd **or more**	1290(19.1)	416(21.0)	433(25.6)	104(6.6)
**Wealth quintiles**				
Poorest	1440(21.3)	417(21.2)	451(26.7)	405(25.8)
Poorer	1563(23.1)	432(21.9)	379(22.5)	377(24.0)
Middle	1434(21.2)	424(21.5)	337(19.9)	301(19.2)
Richer	1250(18.5)	360(18.3)	318(18.8)	227(14.5)
Richest	1074(15.9)	339(17.2)	205(12.1)	260(16.5)
**Covered by health insurance**				
No	6624 (98.0)	1875(95.0)	1673(99.0)	1537(97.9)
Yes	137 (2.0)	98(5.0)	17(1.0)	33.2(2.1)
**Husband level of education**				
No education	2515(37.2)	1396(70.8)	1254(74.2)	103(6.6)
Primary	871(12.9)	218(11.1)	114(6.8)	583(37.2)
Secondary	2311(34.2)	265(13.4)	210(12.4)	749(47.5)
Higher	1063(15.7)	94(4.8)	111(6.6)	138(8.8)
**Difference in age between husband and wife**				
Wife older or same age	104(1.5)	34(1.7)	25(1.5)	77(4.9)
Husband 1–5 years older	1627(24.1)	417(21.1)	273(16.2)	849(54.1)
Husband 6–10 years older	2445(36.2)	663(33.6)	530(31.3)	480(30.6)
Husband > 10 years older	2585(38.2)	860(43.6)	862(51.0)	164(10.5)

### Prevalence of ≥ 8 ANC visits and early timing of first ANC visit

The distribution and prevalence (95% CI) of women attending at least 8 ANC visit and women who had their first ANC visit in the first 3 months of gestation were presented by barriers to healthcare services indicators in Table 3. In all countries, a larger proportion of women considered getting permission to visit the health facility as not a big problem. In terms of getting money needed for treatment, most of the women in Nigeria, Mali and Zambia considered that as not a big problem (53.1, 57.5 and 77.5% respectively), whereas most of the women in Guinea considered getting money for treatment as a big problem (63.6%). Similarly, while the majority of women in Guinea considered distance to the health facility as a big problem (50.8%), most of women in Nigeria, Mali and Zambia considered that as not a big problem (71.5, 69.7 and 62.8% respectively). Women in all the four countries considered not wanting to go to the health facility alone as not a big a problem. The prevalence (95% CI) of at least 8 ANC visits ranged from 1.5 (95% CI: 0.9–2.4) in Zambia to 17.8 (95% CI: 16.4–19.3) in Nigeria. Similarly, early timing of first ANC visits ranged from 17.6 (95% CI: 16.3–18.9) in Nigeria to 38.5 (95% CI: 35.0–41.9) in Zambia (see Table 3).

**Table 3:** Distribution and prevalence of at least 8 ANC visits and early timing of first ANC visit by barriers to healthcare access in Nigeria, Mali, Guinea and Zambia (DHS 2018)

Variable	Nigeria (***N***=6761)			Mali (***N***=1973)			Guinea (***N***=1690)			Zambia (***N***=1570)		
	n(%)	visit >=8	<=3 months	n(%)	visit >=8	<=3 months	n(%)	visit >=8	<=3 months	n(%)	visit >=8	<=3 months
		Prevalence, % (95% CI)	Prevalence, % (95% CI)		Prevalence, % (95% CI)	Prevalence, % (95% CI)		Prevalence, % (95% CI)	Prevalence, % (95% CI)		Prevalence, % (95% CI)	Prevalence, % (95% CI)
		***17.8(16.4–19.3)***	***17.6(16.3–18.9)***		***3.5(2.7–4.6)***	***35.5(32.8–38.3)***		***2.8(2.1–3.9)***	***26.3(23.5–29.4)***		***1.5(0.9–2.4)***	***38.5(35.0–42.1)***
**Getting permission to visit the health facility**		*p* = 0.318	*p* = 0.059		*p*= 0.003	*p*< 0.001		*p*=0.006	*p*=0.002		*p*=0.223	*p*=0.034
Big Problem	757(11.2)	0.16(0.12–0.20)	0.14(0.02–0.18)	570(28.9)	0.02(0.01–0.03)	0.28(0.23–0.33)	494(29.2)	0.02(0.01–0.03)	0.20(0.16–0.25)	88(5.6)	0.03(0.01–0.10)	0.50(0.38–0.61)
Not a big problem	6004(88.8)	0.18(0.16–0.20)	0.18(0.17–0.19)	1403(71.1)	0.04(0.03–0.06)	0.39(0.36–0.42)	1196(70.8)	0.04(0.03–0.05)	0.29(0.26–0.32)	1482(94.4)	0.02(0.01–0.03)	0.38(0.34–0.41)
**Getting money needed for treatment**		p< 0.001	*p*< 0.001		*p*< 0.001	*p*< 0.001		*p*=0.068	*p*=0.143		*p*=0.411	*p*=0.340
Big Problem	3169(46.9)	0.14(0.12–0.16)	0.15(0.14–0.17)	838(42.5)	0.02(0.01–0.03)	0.27(0.23–0.31)	1076(63.6)	0.02(0.01–0.03)	0.25(0.22–0.29)	358(22.5)	0.02(0.01–0.05)	0.41(0.34–0.41)
Not a big problem	3592(53.1)	0.21(0.19–0.23)	0.20(0.18–0.21)	1135(57.5)	0.05(0.04–0.07)	0.42(0.39–0.45)	614(36.4)	0.03(0.02–0.04)	0.29(0.25–0.33)	1217(77.5)	0.02(0.01–0.03)	0.38(0.35–0.42)
**Distance to health facility**		*P* = 0.005	*P* = 0.014		*P*= 0.001	p< 0.001		p< 0.001	*p*=0.007		*p*=0.257	*p*=0.056
Big Problem	1929(28.5)	0.15(0.13–0.17)	0.15(0.13–0.17)	596(30.3)	0.02(0.01–0.03)	0.28(0.24–0.32)	858(50.8)	0.02(0.1–0.03)	0.23(0.19–0.26)	584(37.2)	0.02(0.01–0.04)	0.42(0.37–0.48)
Not a big problem	4832(71.5)	0.19(0.17–0.21)	0.19(0.17–0.20)	1374(69.7)	0.05(0.03–0.06)	0.39(0.36–0.42)	832(49.2)	0.05(0.03–0.07)	0.30(0.26–0.35)	986(62.8)	0.02(0.01–0.03)	0.36(0.32–0.40)
**Not wanting to go alone**		*P* = 0.261	*P* = 0.208		*P*= 0.051	P=0.002		*p*= 0.013	*p*=0.022		*P*=0.448	*p*=0.158
Big Problem	1106(16.4)	0.16(0.14–0.19)	0.16(0.13–0.19)	427(21.6)	0.02(0.01–0.04)	0.28(0.23–0.33)	542(32.1)	0.02(0.01–0.03)	0.22(0.18–0.27)	240(15.3)	0.02(0.01–0.03)	0.44(0.36–0.52)
Not a big problem	5655(83.7)	0.18(0.16–0.19)	0.18(0.17–0.19)	1546(78.4)	0.04(0.03–0.05)	0.38(0.35–0.41)	148(67.9)	0.04(0.03–0.05)	0.28(0.25–0.32)	1330(84.7)	0.02(0.01–0.03)	0.38(0.34–0.41)

### Factors associated with ≥ 8 ANC visits

As shown in the adjusted models, women who considered getting money needed for treatment as not a big problem in Nigeria were more likely to have the recommended number of ANC visits (aOR = 1.38, 95% CI= 1.11–1.73). On the contrary, in Guinea, Zambia and Mali, none of these barriers reduced the odds of having ≥ 8 ANC visits. In terms of the covariates, women in all other age categories in Nigeria were more likely to have the recommended number of ANC visits compared to those aged 19 years or below. Age had no significant association with the recommended number of ANC visits in Guinea, Mali and Zambia. Place of residence also showed significant association with the recommended number of ANC visits only in Nigeria, with women in rural areas less likely to have the recommended number of ANC visits compared to those in urban areas (aOR = 0.24, 95% CI = 0.14–0.42). Religion had significant association with having ≥ 8 ANC visits among women in Nigeria and Guinea, with Muslim women less likely to have ≥ 8 ANC visits compared to Christians (aOR = 0.44, 95% CI = 0.34–0.56) and (aOR=0.24, 95% CI = 0.11–0.52 respectively).

In all the four countries, the odds of having ≥ 8 ANC visits among women decreased with higher birth order, with women with five or more birth order less likely to have the recommended number of ANC visits. Polygyny showed significant association with ≥ 8 ANC visits in Nigeria and Zambia. Wealth quintile was only associated with ≥ 8 ANC visits in Nigeria, with women in the middle (aOR = 2.35, 95% CI = 1.50–3.69), richer (aOR = 3.35, 95% CI = 2.08–5.38) and richest (aOR = 4.77 95% CI = 2.91–7.82) wealth quintile, more likely to have ≥ 8 ANC visits. Health insurance coverage also showed association with ≥ 8 ANC visits only among women in Zambia and the odds of having ≥ 8 ANC visits increased among women who had health insurance (aOR = 8.27 95% CI 1.34–51.01). In Nigeria, Mali and Zambia, the likelihood of ≥ 8 ANC visits was high among women whose husbands had at least primary education, with higher odds among those whose husbands had higher education (see Table 4).

**Table 4:** Odd ratios and 95% CI of factors associated with ≥ 8 ANC visits in Nigeria, Mali, Guinea and Zambia (DHS 2018)

Variable	Nigeria (*N*=6761)		Mali (*N*=1973)		Guinea (*N*=1690)		Zambia (*N*=1570)	
	cOR(95% CI)	aOR(95% CI)	cOR(95% CI)	aOR (95% CI)	cOR(95% CI)	aOR(95% CI)	cOR(95% CI)	aOR (95% CI)
**Getting permission to visit the health facility**								
Big problem	Reference (1.0)	Reference (1.0)	Reference (1.0)	Reference (1.0)	Reference (1.0)	Reference (1.0)	Reference (1.0)	Reference (1.0)
Not a big problem	1.16(0.47–0.91)	0.71(0.51–1.02)	3.89(1.50–10.11)***	2.20(0.61–7.91)	3.49(1.35–8.99)**	2.99(0.89–10.01)	0.43(0.11–1.73)	0.22(0.06–0.79)**
**Getting money needed for treatment**								
Big problem	Reference (1.0)	Reference (1.0)	Reference (1.0)	Reference (1.0)	Reference (1.0)	Reference (1.0)	Reference (1.0)	Reference (1.0)
Not a big problem	1.38(1.10–1.72)**	1.38(1.11–1.73)**	3.75(1.92–7.33)***	1.33(0.52–3.44)	1.89(0.94–3.79)	0.54(0.22–1.29)	0.63(00.21–1.92)	0.97(0.34–2.79)
**Distance to health facility**								
Big Problem	Reference (1.0)	Reference (1.0)	Reference (1.0)	Reference (1.0)	Reference (1.0)	Reference (1.0)	Reference (1.0)	Reference (1.0)
Not a big problem	0.66(0.51–0.86)**	0.73(0.55–0.97)**	3.55(1.56–8.04)**	1.85(0.87–3.93)	4.86(2.21–10.8)***	2.96(0.96–9.07)	0.61(0.26–1.45)	0.25(0.06–1.06)
**Not wanting to go alone**								
Big Problem	Reference (1.0)	Reference (1.0)	Reference (1.0)	Reference (1.0)	Reference (1.0)	Reference (1.0)	Reference (1.0)	Reference (1.0)
Not a big problem	1.14(0.91–1.44)	0.77(0.57–1.04)	2.22(0.98–5.07)	0.42(0.20–0.90)**	2.91(1.21–699)**	0.72(0.23–2.28)	1.64(0.45–6.03)	2.58(0.55–12.00)
**Age at child birth (years)**								
≤19	Reference (1.0)	Reference (1.0)	Reference (1.0)	Reference (1.0)	Reference (1.0)	Reference (1.0)	Reference (1.0)	Reference (1.0)
20–24	1.62(1.09–2.39)**	1.61(1.09–2.38)**	0.80(0.36–1.79)	0.72(0.30–1.70)	1.54(0.62–3.83)	1.59(0.64–3.98)	0.51(0.14–1.86)	0.42(0.13–1.34)
25–29	2.26(1.50–3.41)***	2.26(1.50–3.40)***	0.58(0.27–1.24)	0.68(0.27–1.76)	1.62(0.63–4.14)	2.41(0.84–6.87)	0.24(0.06–0.91)*	0.55(0.11–2.78)
30–34	3.04(1.96–4.70)***	3.02(1.95–4.67)***	0.38(0.15–0.97)**	0.59(0.16–2.14)	1.31(0.47–3.63)	2.29(0.62–8.43)	0.49(0.11–2.18)	1.88(0.55–6.42)
35–39	2.92(1.82–4.68)***	2.90(1.81–4.64)***	0.73(0.27–1.93)	1.78(0.43–7.41)	0.33(0.06–1.70)	1.30(0.117–9.79)	0.02(0.03–0.19)**	0.26(0.02–4.15)
≥40	3.01(1.67–5.40)***	3.00(1.67–5.39)***	0.35(0.07–1.79)	1.15(0.14–9.29)	0.71(0.14–3.70)	3.74(0.43–32.48)	0.46(0.09–2.38)	17.94(2.94–109.29)**
**Place of residence**								
Urban	Reference (1.0)	Reference (1.0)	Reference (1.0)	Reference (1.0)	Reference (1.0)	Reference (1.0)	Reference (1.0)	Reference (1.0)
Rural	0.24(0.20–0.29)***	0.24(0.14–0.42)***	0.25(0.14–0.45)***	0.87(0.40–1.88)	0.14(0.07–0.28)***	0.56(0.20–1.58)	0.90(0.32–2.54)	0.89(0.19–4.11)
**Religion**								
Christians	Reference (1.0)	Reference (1.0)	Reference (1.0)	Reference (1.0)	Reference (1.0)	Reference (1.0)	Reference (1.0)	Reference (1.0)
Muslim	0.20(0.16–0.24)***	0.44(0.34–0.56)***	1.83(0.27–12.55)	2.86(0.46–17.70)	0.45(0.20–0.99)***	0.24(0.11–0.52)***	2.44(0.27–21.80)	4.59(0.56–37.99)
Others	0.18(0.03–0.91)**	0.30(0.07–1.64)	0.83(0.05–12.82)	3.56(0.20–62.39)	1.00(0.33–3.02)	0.85(0.19–3.89)	0.42(0.05–3.40)	0.40(0.03–5.62)
**Birth order**								
1–2	Reference (1.0)	Reference (1.0)	Reference (1.0)	Reference (1.0)	Reference (1.0)	Reference (1.0)	Reference (1.0)	Reference (1.0)
3–4	0.75(0.62–0.90)**	0.67(0.54–0.84)***	0.65(0.34–1.24)	0.79(0.36–1.76)	0.94(0.51–1.72)	0.77(0.48–1.58)	0.13(0.03–0.55)**	0.04(0.10–0.20)***
≥5	0.36(0.29–0.44)***	0.42(0.30–0.60)***	0.22(0.10–0.48)***	0.25(0.06–0.98)**	0.18(0.07–0.51)***	0.23(0.06–0.88)**	0.09(0.02–0.36)**	0.01(0.002–0.07)***
**Pregnancy intention**								
No (later/no more)	Reference (1.0)	Reference (1.0)	Reference (1.0)	Reference (1.0)	Reference (1.0)	Reference (1.0)	Reference (1.0)	Reference (1.0)
Yes (then)	0.68(0.55–0.85)**	1.04(0.79–1.35)	1.23(0.56–2.73)	0.94(0.40–2.18)	3.70(1.06–12.86)**	3.13(0.84–11.66)	0.91(0.35–2.36)	0.58(0.22–1.54)
**Polygyny**								
Monogamous	Reference (1.0)	Reference (1.0)	Reference (1.0)	Reference (1.0)	Reference (1.0)	Reference (1.0)	Reference (1.0)	Reference (1.0)
Polygamous as first wife	0.23(0.15–0.34)***	0.67(0.43–1.05)	0.55(0.23–1.32)	1.51(0.49–4.66)	0.14(0.02–1.04)	0.26(0.03–2.30)	4.42(0.94–2.89)	40.35(6.65–244.75)
Polygamous as 2nd **or** higher	0.37(0.29–0.46)***	0.93(0.71–1.23)	1.01(0.50–2.03)	1.79(0.81–3.96)	1.07(0.48–2.40)	1.44(0.57–3.65)	11.32(3.33–18.30)***	15.6(0.50–23.10)***
**Wealth quintiles**								
Poorest	Reference (1.0)	Reference (1.0)	Reference (1.0)	Reference (1.0)	Reference (1.0)	Reference (1.0)	Reference (1.0)	Reference (1.0)
Poorer	2.01(1.32–3.07)**	1.54(1.00–2.06)	1.23(0.27–5.51)	1.10(0.24–5.06)	0.32(0.04–2.89)	0.29(0.03–2.73)	1.78(0.41–7.8)	0.88(0.22–3.52)
Middle	4.57(3.01–6.95)***	2.35(1.50–3.69)***	1.38(0.33–5.77)	1.09(0.22–5.41)	1.75(0.49–6.23)	1.17(0.31–4.38)	1.60(0.41–6.3)	0.95(0.24–3.81)
Richer	9.71(6.38–14.79)***	3.35(2.08–5.38)***	3.31(0.90–12.14)	1.82(0.37–9.08)	5.11(1.65–15.82)**	1.90(0.48–7.52)	0.93(0.17–5.06)	0.56(0.09–3.64)
Richest	21.31(14.06–32.29)***	4.77(2.91–7.82)***	11.40(3.29–39.53)***	4.00(0.68–23.36)	11.46(4.15–31.63)***	3.28(0.77–13.98)	2.82(0.67–11.80)	0.58(0.07–4.68)
**Covered by Health Insurance**								
No	Reference (1.0)	Reference (1.0)	Reference (1.0)	Reference (1.0)	Reference (1.0)	Reference (1.0)	Reference (1.0)	Reference (1.0)
Yes	3.27(2.15–4.98)***	1.36(0.86–2.16)	6.30(2.88–13.80)***	1.68(0.63–4.52)	2.11(0.26–16.94)	0.50(0.04–6.55)	9.36(3.87–53.67)**	8.27(1.34–51.01)**
**Husband level of education**								
No education	Reference (1.0)	Reference (1.0)	Reference (1.0)	Reference (1.0)	Reference (1.0)	Reference (1.0)	Reference (1.0)	Reference (1.0)
Primary	4.28(3.04–6.03)***	2.06(1.41–3.09)***	2.67(1.24–5.74)**	1.96(0.88–4.36)	0.57(0.12–2.59)	0.47(0.09–2.33)	3.88(0.42–35.44)	1.14(0.10–13.32)
Secondary	7.79(5.73–10.60)***	2.04(1.43–2.92)***	4.34(2.18–8.65)***	1.97(0.84–4.61)	2.11(0.90–4.91)	1.21(0.48–3.11)	10.51(1.34–82.3)**	5.54(0.65–47.35)
Higher	10.59(7.60–14.75)***	2.09(1.41–3.08)***	12.77(5.28–30.90)***	3.49(1.16–10.49)*	4.49(1.90–10.62)**	1.18(0.48–2.91)	33.49(3.62–309.03)**	18.67(1.31–267.00)**
**Difference in age between husband and wife**								
Wife older or same age	Reference (1.0)	Reference (1.0)	Reference (1.0)	Reference (1.0)	5.36(0.09–15.2)***	13.3(5.93–48.7)***	Reference (1.0)	Reference (1.0)
Husband 1–5 years older	1.01(0.61–1.71)	1.29(0.75–2.22)	0.40(0.11–1.41)	0.43(0.10–1.86)	0.96(0.43–2.17)	1.80(0.402.95)	1.39(0.29–6.77)	0.69(0.10–4.64)
Husband 6–10 years older	0.60(0.35–1.02)	1.05(0.60–1.84)	0.31(0.08–1.13)	0.33(0.08–1.36)	0.82(0.42–1.61)	0.86(0.39–1.90)	0.86(0.16–4.70)	0.46(0.06–3.26)
Husband > 10 years older	0.40(0.23–0.70)**	0.97(0.55–1.74)	0.36(0.11–1.21)	0.32(0.08–1.30)	Reference (1.0)	Reference (1.0)	0.52(0.05–5.98)	0.50(0.05–5.43)

***p< 0.001,**p< 0.05,*p< 0.10

#### Factors associated with early timing of first ANC visit

In the adjusted models, getting permission to visit the health facility (aOR = 1.62, 95% CI = 1.15–2.33) motivated women to have early timing of ANC visits in Guinea whiles getting money needed for treatment (aOR = 1.38, 95% CI = 1.03–1.92) influenced early timing of ANC visits in Mali. In terms of the covariates, place of residence showed significant association with early timing of ANC visits in Mali and Zambia, where early timing of ANC visits was high among women who lived in rural areas (aOR = 1.53, 95% CI = 1.03–2.26 and aOR = 1.99, 95% CI = 1.29–3.08). Religion showed significant association with early timing of ANC visits in Nigeria and Guinea. However, whereas Muslim women in Nigeria were less likely to have early timing of ANC visits (aOR = 0.56, 95% CI = 0.44–0.71), those in Guinea were more likely to have early timing of ANC visits (aOR = 3.80, 95% CI = 1.94–7.45).

In Nigeria and Mali, the odds of having early timing of ANC visits decreased with higher birth order, with women with five or more birth order less likely to have the recommended number of ANC visits as shown in Table 5 (aOR = 0.56, 95% CI = 0.41–0.78 and aOR = 0.61, 95% CI = 0.40–0.93 respectively). Pregnancy intention also showed significant association with early timing of ANC visits only in Nigeria as women who wanted their pregnancy at the time they were pregnant more likely to have early timing of ANC visits (aOR = 1.54, 95% CI = 1.19–1.99 and aOR = 1.70, 95% CI = 1.27–2.29 respectively). Wealth quintile had association with early timing of ANC in Nigeria, Mali and Guinea, with high odds among women in the richest wealth quintile (aOR = 3.21, 95% CI = 2.12–4.85; aOR = 4.04, 95% CI = 2.49–6.59; aOR = 3.55, 95% CI = 1.87–6.73 respectively).

**Table 5:** Odd ratios and 95% CI of factors associated with early timing of ANC visits (<= 3 months gestation) in Nigeria, Mali, Guinea and Zambia (DHS 2018)

Variable	Nigeria (*N*=6709)		Mali (*N*=1973)		Guinea (*N*=1690)		Zambia (*N*=1570)	
	cOR (95% CI)	aOR (95% CI)	aOR (95% CI)	aOR (95% CI)	cOR (95% CI)	aOR (95% CI)	aOR(95% CI)	aOR (95% CI)
**Getting permission to visit the health facility**								
Big problem	Reference (1.0)	Reference (1.0)	Reference (1.0)	Reference (1.0)	Reference (1.0)	Reference (1.0)	Reference (1.0)	Reference (1.0)
Not a big problem	1.32(0.99–1.18)	1.02(0.74–1.39)	1.62(1.25–2.12)***	1.05(0.73–1.52)	1.63(1.20–2.22)**	1.62(1.15–2.33)**	0.62(0.40–0.97)**	0.67(0.42–1.07)
**Getting money needed for treatment**								
Big problem	Reference (1.0)	Reference (1.0)	Reference (1.0)	Reference (1.0)	Reference (1.0)	Reference (1.0)	Reference (1.0)	Reference (1.0)
Not a big problem	1.36(1.16–1.59)***	1.14(0.95–1.37)	1.94(1.55–2.43)***	1.38(1.03–1.92)**	1.20(0.94–1.54)	0.77(0.58–1.03)	0.87(0.65–1.16)	1.10(0.80–1.51)
**Distance to health facility**								
Big problem	Reference (1.0)	Reference (1.0)	Reference (1.0)	Reference (1.0)	Reference (1.0)	Reference (1.0)	Reference (1.0)	Reference (1.0)
Not a big problem	1.26(1.04–1.52)**	0.90(0.70–1.14)	1.67(1.31–2.12)***	1.04(0.71–1.51)	1.48(1.11–1.98)**	1.08(0.73–1.60)	0.78(0.61–1.01)	0.94(0.69–1.27)
**Not wanting to go alone**								
Big Problem	Reference (1.0)	Reference (1.0)	Reference (1.0)	Reference (1.0)	Reference (1.0)	Reference (1.0)	Reference (1.0)	Reference (1.0)
Not a big problem	1.16(0.92–1.45)	0.90(0.69–1.18)	1.57(1.18–2.10)**	0.87(0.58–1.32)	1.39(1.05–1.84)**	1.06(0.73–1.53)	0.77(0.54–1.11)	1.02(0.66–1.57)
**Age at child birth (years)**								
≤19	Reference (1.0)	Reference (1.0)	Reference (1.0)	Reference (1.0)	Reference (1.0)	Reference (1.0)	Reference (1.0)	Reference (1.0)
20–24	1.33(1.02–1.74)**	1.20(0.90–1.58)	0.99(0.71–1.37)	1.21(0.84–1.74)	1.33(0.87–2.04)	1.31(0.83–2.08)	1.09(0.72–1.65)	1.13(0.73–1.75)
25–29	1.42(1.08–1.86)**	1.32(0.96–1.82)	0.79(0.55–1.12)	1.21(0.78–1.86)	1.17(0.80–1.72)	1.19(0.75–1.88)	0.73(0.47–1.12)	0.86(0.49–1.53)
30–34	1.46(1.09–1.95)**	1.48(0.98–2.24)	0.65(0.45–0.95)**	1.17(0.69–2.01)	1.39(0.94–2.07)	1.55(0.94–2.56)	0.71(0.46–1.10)	0.96(0.51–1.78)
35–39	1.13(0.82–1.56)	1.33(0.88–2.01)	0.84(0.55–1.27)	1.69(0.95–3.01)	1.04(0.67–1.61)	1.13(0.63–2.02)	0.51(0.29–0.91)**	0.74(0.38–1.44)
≥40	0.72(0.47–1.10)	1.11(0.65–1.90)	0.51(0.29–0.92)**	1.10(0.55–2.22)	0.93(0.48–1.79)	0.99(0.40–2.45)	0.64(0.37–1.11)	0.81(0.36–1.79)
**Residence**								
Urban	Reference (1.0)	Reference (1.0)	Reference (1.0)	Reference (1.0)	Reference (1.0)	Reference (1.0)	Reference (1.0)	Reference (1.0)
Rural	0.55(0.46–0.66)***	0.34(0.83–1.24)	0.43(0.33–0.55)***	1.53(1.03–2.26)**	0.53(0.49–0.74)***	1.30(0.81–2.08)	1.75(1.30–2.42)***	1.99(1.29–3.08)**
**Religion**								
Christians	Reference (1.0)	Reference (1.0)	Reference (1.0)	Reference (1.0)	Reference (1.0)	Reference (1.0)	Reference (1.0)	Reference (1.0)
Muslim	0.35(0.29–0.42)***	0.56(0.44–0.71)***	1.12(0.65–1.93)	1.30(0.71–2.37)	4.04(2.04–8.03)***	3.80(1.94–7.45)***	0.76(0.18–3.12)	0.73(0.17–3.06)
Others	0.25(0.05–1.22)	0.35(0.08–1.62)	0.68(0.32–1.43)	1.32(0.61–2.82)	1.63(0.53–5.02)	1.53(0.50–4.68)	1.30(0.34–4.99)	1.60(0.35–7.31)
**Birth order**								
1–2	Reference (1.0)	Reference (1.0)	Reference (1.0)	Reference (1.0)	Reference (1.0)	Reference (1.0)	Reference (1.0)	Reference (1.0)
3–4	0.74(0.62–0.88)**	0.76(0.61–0.94)**	0.68(0.53–0.88)**	0.73(0.53–1.01)	0.95(0.73–1.25)	0.93(0.66–1.30)	0.73(0.56–0.95)**	0.78(0.56–1.009)
≥5	0.42(0.34–0.51)***	0.56(0.41–0.78)**	0.46(0.35–0.59)***	0.61(0.40–0.93)**	0.96(0.73–1.25)	1.07(0.69–1.66)	0.61(0.45–0.84)**	0.66(0.39–1.11)
**Pregnancy intention**								
No (later/no more)	Reference (1.0)	Reference (1.0)	Reference (1.0)	Reference (1.0)	Reference (1.0)	Reference (1.0)	Reference (1.0)	Reference (1.0)
Yes (then)	1.13(0.88–1.46)	1.54(1.19–1.99)**	1.77(1.33–2.37)***	1.70(1.27–2.29)***	1.10(0.79–1.54)	1.10(0.78–1.54)	1.08(0.83–1.40)	1.00(0.77–1.30)
**Polygyny**								
Monogamous	Reference (1.0)	Reference (1.0)	Reference (1.0)	Reference (1.0)	Reference (1.0)	Reference (1.0)	Reference (1.0)	Reference (1.0)
Polygamous as first wife	0.42(0.31–0.56)***	0.88(0.64–1.20)	0.51(0.36–0.71)***	0.73(0.49–1.07)	0.90(0.59–1.34)	0.96(0.62–1.50)	1.23(0.62–2.46)	1.78(0.76–1.30)
Polygamous as 2nd or higher	0.54(0.43–0.68)***	0.89(0.69–1.15)	1.05(0.83–1.33)	1.29(0.99–1.69)	0.88(0.64–1.22)	0.85(0.60–1.20)	1.23(0.63–2.39)	1.19(0.60–2.35)
**Wealth quintiles**								
Poorest	Reference (1.0)	Reference (1.0)	Reference (1.0)	Reference (1.0)	Reference (1.0)	Reference (1.0)	Reference (1.0)	Reference (1.0)
Poorer	2.02(1.54–2.65)***	1.70(1.29–2.25)***	1.145(0.78–1.68)	1.13(0.76–1.67)	1.65(1.12–2.32)**	1.62(1.1–2.37)**	0.70(0.51–0.96)**	0.72(0.52–1.01)
Middle	2.85(2.16–3.78)***	2.01(1.46–2.76)***	1.62(1.14–2.29)**	1.51(1.05–2.18)**	2.02(1.32–3.08)**	1.99(1.29–3.07)**	0.57(0.40–0.80)**	0.73(0.51–1.04)
Richer	3.35(2.53–4.43)***	1.99(1.41–2.81)***	2.48(1.75–3.53)***	2.01(1.35–3.00)**	2.93(1.93–4.46)***	2.68(1.62–4.44)**	0.48(0.31–0.74)**	0.83(0.48–1.44)
Richest	6.65(4.99–8.87)***	3.21(2.12–4.85)***	5.53(3.79–8.06)***	4.04(2.28–7.17)***	4.05(2.49–6.59)***	3.55(1.87–6.73)**	0.64(0.44–0.94)**	1.22(0.67–2.23)
**Covered by Health Insurance**								
No	Reference (1.0)	Reference (1.0)	Reference (1.0)	Reference (1.0)	Reference (1.0)	Reference (1.0)	Reference (1.0)	Reference (1.0)
Yes	1.59(0.98–2.57)	0.88(0.53–1.47)	5.24(2.83–9.71)***	1.77(0.92–3.39)	2.10(0.67–6.52)	0.92(0.26–3.30)	0.81(0.32–2.04)	0.71(0.26–1.97)
**Husband level of education**								
No education	Reference (1.0)	Reference (1.0)	Reference (1.0)	Reference (1.0)	Reference (1.0)	Reference (1.0)	Reference (1.0)	Reference (1.0)
Primary	2.32(1.72–3.13)***	1.60(1.16–2.21)**	1.85(1.37–2.51)***	1.56(1.11–2.18)**	0.96(0.56–1.65)	0.85(0.50–1.47)	0.83(0.49–1.41)	0.79(0.47–1.35)
Secondary	3.52(2.70–4.58)***	1.72(1.24–2.38)**	3.02(2.21–4.10)***	1.88(1.30–2.72)**	1.52(1.09–2.11)**	1.32(0.93–1.86)	0.68(0.39–1.17)	0.71(0.41–1.22)
Higher	3.68(2.73–4.96)***	1.56(1.05–2.31)**	8.38(4.18–16.79)***	3.21(1.52–6.78)**	2.71(1.74–4.23)***	1.94(1.23–3.07)**	0.92(0.48–1.75)	1.06(0.49–2.33)
**Difference in age between husband and wife**								
Wife older or same age	Reference (1.0)	Reference (1.0)	Reference (1.0)	Reference (1.0)	0.94(0.38–2.36)	Reference (1.0)	Reference (1.0)	Reference (1.0)
Husband 1–5 years older	1.41(0.77–2.59)	1.55(0.82–2.95)	0.46(0.23–0.94)**	0.48(0.23–0.99)**	0.79(0.55–1.15)	0.98(0.35–2.79)	1.80(0.94–3.43)	1.51(0.87–2.66)
Husband 6–10 years older	1.07(0.60–1.94)	1.49(0.80–2.75)	0.53(0.26–1.07)**	0.58(0.28–1.21)	0.84(0.63–1.11)	0.77(0.51–1.15)	1.51(0.78–2.91)	1.39(0.78–2.48)
Husband > 10 years older	0.95(0.53–1.71)	1.67(0.90–3.10)	0.61(0.31–1.21)	0.61(0.30–1.26)	Reference (1.0)	0.80(0.60–1.09)	2.17(1.06–4.48)	2.02(1.05–3.86)**

***p< 0.001,**p< 0.05,*p< 0.10

Apart from Zambia, husband’s level of education had an association with early timing of ANC visits in the rest of the countries and early timing of ANC visits increased with higher husband’s level of education. Difference in age between husband and wife was significant with early timing of ANC visits in Mali and Zambia. However, while women whose husbands were 1–5 years older than them were less likely to have early timing of ANC visits in Mali, those whose husbands were more than 10 years older than them were more likely to have early timing of ANC visits in Zambia, compared to those who were either older or of the same age as their husbands.

## Discussion

This study examined the barriers to healthcare access and utilization of ANC services in Nigeria, Mali, Guinea and Zambia using data from recent DHS in SSA. We found that the prevalence of at least 8 ANC visits was low in Zambia but high Nigeria. Women who considered getting money needed for treatment as not being a big problem in Nigeria were more likely to have the recommended number of ANC visits. On the contrary, in Guinea, Zambia and Mali, none of these barriers reduced the odds of having ≥ 8 ANC visits. Getting permission to visit the health facility motivated women to have early ANC visits in Guinea while getting money needed for treatment influenced early timing of ANC visits in Mali. In terms of the covariates, age at childbirth, place of residence, religion, birth order, pregnancy, polygyny, wealth quintile, health insurance, husband’s level of education and difference in age between husband and wife showed significant associations with ≥ 8 ANC visits and early timing of first ANC visit in either of the four countries.

Our study revealed that, compared to women in Zambia, those in Nigeria had the highest prevalence of ≥ 8 ANC visits. However, early timing of first ANC visit was high in Zambia compared to Nigeria. Previous studies in Nigeria have identified high prevalence of ANC attendance among women [30–32]. These studies reported higher prevalence of ANC visits, compared to what was found in this study. The possible reason for the finding is differences in the cut-off for recommended number of ANC visits. While these studies used at least 4 ANC visits, the cut-off in this study was at least 8 ANC visits based on the WHO recent guidelines on recommended ANC visits. Despite the high prevalence of ANC visits in Nigeria, our finding that Nigeria had the least prevalence in terms of early timing of first ANC visit is in line with the findings of previous studies that have been conducted in the country [10, 33]. Having primary education, living in rural areas and not having health insurance were cited as some of the reasons for delayed ANC visits among women in Nigeria [33]. Our findings that women in Zambia have low prevalence of at least 8 ANC visits but high prevalence of early ANC visits contradicts several studies which have been carried out in the country. Most of the previous studies found more women to have at least four ANC visits but low prevalence of early ANC visits [34–36]. The possible reason for the finding could be the disparities in cut-off of both recommended number of ANC visits and early timing of first ANC visit in our study, compared to previous studies.

As found in this study, getting money needed for treatment was the only barrier to having at least 8 ANC visits in Nigeria. Similar barrier has been found to be associated with ANC attendance in Nigeria in previous studies [31, 32, 37]. This factor has been explained to be related to cost, place of residence and spousal support for ANC attendance. The possible reason for these barriers reducing the odds of women having at least 8 ANC visits could be that the lack of spousal support and financial and geographic empowerment serve as hindrance to ANC access. In line with this, low socio-economic status of the women, in terms of poor wealth quintile, no formal education of the husband and rural dwelling have been identified as factors that reduce the likelihood of having at least 8 ANC visits in Nigeria [31, 38–41]. However, in the adjusted model, none of these barriers predicted early timing of ANC visits in the country. A more exploratory study using a qualitative research approach can help find out some reasons behind this finding.

In Mali, getting money needed for treatment was a barrier to early timing of ANC visits. Similarly, previous studies in Mali found that money was the main problem for both number and timing of ANC visits. For instance, a systematic review on determinants of ANC utilization in SSA, that included Mali identified cost as one of the barriers associated with ANC utilization in SSA [10]. In terms of the covariates, age, place of residence, birth order, pregnancy, polygyny, wealth quintile, health insurance, husband’s level of education and difference in age between husband and wife showed significant associations with ≥ 8 ANC visits and early timing of first ANC in Mali. These factors have been found in previous studies carried out in Mali to have associations with the number and timing of ANC visits [42, 43]. Most of these factors play a role in determining the socio-economic status of the woman, and consequently her ability to access ANC services. For instance, as found in this study, women whose husbands had secondary education and those who lived in urban areas, those who had health insurance and had higher wealth status were more likely to utilize ANC services. Such socio-economic empowerment measures can play a role in reducing some of the barriers to having more ANC visits and early timing of ANC visits.

We found that in Guinea, not getting permission to visit the health facility was a barrier to early timing of first ANC visits. This finding operated through significant socio-economic covariates (wealth quintile and husband’s educational level), which showed positive associations with early timing of first ANC visits in the country. The possible reason for this finding could be the socio-economic disparity with access to maternal healthcare services, including ANC in Guinea as the country is still recovery from the impact of the Ebola virus disease on maternal and child health services [44–46]. Consistent with the findings of previous studies in Zambia [34–36], this study revealed that none of the four barriers influenced having at least 8 ANC visits and early timing of ANC visits among women in the country. Apart from this finding, this study revealed that women who were covered by health insurance, those with high wealth quintile and those whose husbands had higher levels of education were more likely to either have at least 8 ANC visits or early timing of first ANC visits in Zambia. Similar findings have also been obtained in previous studies that have been carried out in the country [34, 35].

### Strengths and limitations

One of the key strengths of the study lies in the use of the most recent nationally representative datasets of women of reproductive age in four sub-Saharan African countries to examine the influence of barriers to healthcare access and other factors on the WHO recommendation of 8 or more visits and early timing of first antenatal visit. Moreover, the sample size used in this study supports the generalizability of the findings to all women of reproductive age in the four countries. This study has some limitations. First, the cross-sectional nature of the DHS dataset does not allow for causal relationships but only associations between barriers to healthcare access and ANC services utilization. Secondly, as it happens in every study, participation in the surveys is not compulsory and hence some pregnant women may be excluded due to their unwillingness to be part of the survey and this can affect the sample size. Thirdly, this study only considered barriers to healthcare access indicators that were available in the DHS dataset, although there can be other barriers which are not in the DHS dataset which could have significant associations with ANC services utilization. Moreover, in this study, only currently married women were eligible since most of the questions were asked from married women. Also, the order of the antenatal care visit or timing of subsequent visit were not reported in DHS. Finally, both the quality of ANC and previous or current pregnancy care experience from healthcare provider which may influence decisions to attend or use ANC services were not controlled for in this study.

## Conclusion

Our study has emphasized the role barriers to healthcare access play in ANC services utilization across sub-Saharan African countries. Adequate and early ANC visits are essential in ensuring the health of the mother and fetus and prevent pregnancy related complications. Despite the efforts by WHO to reduce mortality related to pregnancy, which has led to increasing the minimum of ANC visits from four to eight contacts, most of the countries in SSA may not have implemented the WHO recommendation. This recommendation is essential for the reduction in childbirth complications, maternal and neonatal mortality rates considering the high burden of neonatal and mortality in the sub-Saharan African region. More importantly, there is the need for governmental and non-governmental organizations to ensure that policies geared towards improving the quality of ANC services and promoting good interaction between health care seekers and health care providers are integrated within the health system. Future studies should explore the barriers towards the full implementation of the WHO ANC model in SSA and to compare experiences of pregnant women who have adhered to the ANC model and those who have not. More importantly future studies should examine the order and timing of subsequent visits among women seeking ANC.

## Data Availability

Data for this study were sourced from Demographic and Health surveys (DHS) and available here: http://dhsprogram.com/data/available-datasets.cfm.

## Abbreviations

ANC: Antenatal care
DHS: Demographic and Health Survey
MDGs: Millennium Development Goals
SDGs: Sustainable Development Goals
SSA: Sub-Saharan Africa
WHO: World Health Organization
